# Angiotensin-converting enzyme inhibitors will help in improving stroke outcome if given immediately after stroke

**DOI:** 10.4103/0972-2327.70869

**Published:** 2010

**Authors:** M. V. Padma

**Affiliations:** Department of Neurology, AIIMS, New Delhi, India

## Introduction

Although there is no controversy regarding the benefits of antihypertensive medication in reducing the risk of first time or recurrent stroke, raging controversy persists regarding the type of antihypertensives best suited as well as the so-called non–blood pressure (BP) lowering beneficial effects of certain “class” of antihypertensives and the effect of antihypertensives given to improve acute stroke outcomes.

The goal of antihypertensive therapy is to reduce cardiovascular and cerebrovascular morbidity and mortality associated with arterial hypertension by a strategy focused on lowering BP, while minimizing the impact of other associated cardiovascular risk factors. The conclusion drawn from the initial studies was that the reduction of cardiovascular events and death observed in hypertensive patients was mainly related to the magnitude of the fall in BP achieved by treatment. Benefit could not be ascribed to a given class of therapy because studies were not designed to compare agents but rather to determine whether active therapy differed from placebo in preventing cardiovascular morbidity and mortality.

For primary prevention, the information from the Heart Outcomes Prevention Evaluation (HOPE) trial,[1] Losartan Intervention For Endpoint reduction to Hypertension (LIFE) trial,[2] Study on Cognition and Prognosis in the Elderly (SCOPE),[3] and Australian National Blood Pressure Study (ANBP)[4] support the view that BP lowering protects against stroke *regardless of baseline blood pressure level*. There is increasing evidence that blockade of the angiotensin system gives additional protection. For secondary prevention, evidence from the Perindopril Protection Against Recurrent Stroke Study (PROGRESS)[5] shows that BP lowering with perindopril-based therapy reduces fatal or nonfatal stroke events, again in hypertensive or normotensive individuals. There is uncertainty about BP lowering in acute stroke, although presentation of the Acute Candesartan Cilexetil Evaluation in Stroke Survivors (ACCESS) trial[6] showed significant protection against vascular events using candesartan, which suggests further studies to be undertaken.

The current review evaluates the role of ACE inhibitors in improving stroke outcomes. Despite the prevalence of arterial hypertension following stroke, its optimal management has not been established.[7–11] An elevated BP can result from the stress of stroke, a full bladder, pain, preexisting hypertension, a physiologic response to hypoxia, or increased intracranial pressure. Theoretical reasons to lower BP include reducing the formation of brain edema, lessening the risk of hemorrhage transformation of infarction, preventing further vascular damage, and forestalling early recurrent stroke. However, aggressive treatment of elevated BP could be detrimental because of secondary reduction of perfusion in the area of ischemia, which could expand the size of the infarction.[7]

Because of these conflicting issues and the lack of unambiguous data, the appropriate treatment of BP in the setting of acute ischemic stroke remains controversial. Although there are no definitive data from controlled clinical trials, in the absence of other organ dysfunction necessitating rapid reduction in BP, or in the setting of thrombolytic therapy there isn’t adequate scientific evidence for lowering BP among patients with acute ischemic stroke.[7] Situations that might require urgent antihypertensive therapy include hypertensive encephalopathy, aortic dissection, acute renal failure, acute pulmonary edema, or acute myocardial infarction.[12] Although severe hypertension might be considered as an indication for treatment, there are no data to define the levels of arterial hypertension that mandate emergent management.[12] The consensus is that antihypertensive agents should be withheld unless the diastolic BP is >120 mmHg or unless the systolic BP is >220 mmHg. There is general agreement to recommend a cautious approach toward the treatment of arterial hypertension in acute setting. Agents that have a short duration of action and little effect on cerebral blood vessels are preferred. Because some patients can have neurologic worsening with rapid lowering of the BP, the use of sublingual nifedipine and other antihypertensive agents causing precipitous reductions in BP should be avoided.

Given this background, we will now review the renin–angiotensin system (RAS), angiotensin-converting enzyme (ACE) inhibition, and the possible beneficial effect of ACE inhibition in acute stroke.[13,14] ACE inhibitors are now being purported as agents that can salvage the acutely jeopardized brain tissue after acute stroke with their non-BP lowering beneficial effects.

## Renin-Angiotensin System and Stroke

The RAS has been implicated in hypertension, as well as in a number of genetic, humoral, and cellular mechanisms that may be involved in atherogenesis or related phenomenon in hypertension. Angiotensin-converting enzyme inhibitors (ACE-Is) were introduced for the treatment of high BP in the 1970s. They act on the RAS by blocking the conversion of angiotensin I to angiotensin II by inhibiting the ACE.

ACE-Is have been shown to block the activation of RAS in plasma as well as in the vascular wall. Their clinical use has been based on the efficacy (which is not different from diuretics and β-blockers), tolerability, and easy combination. Their popularity is also due to the beneficial effects of drugs on intermediate or surrogate end points, such as regression of left ventricular hypertrophy,[13,14] or the ability of drugs to diminish proteinuria.[15,16] Beneficial effects of ACE-Is in secondary prevention after acute myocardial infarction and congestive heart failure, as well as in diabetic and nondiabetic nephropathy[15] have further contributed to the increase in their use.

Physiologic and pathologic studies in hypertensives receiving ACE-Is have shown that vascular compliance is increased after therapy. Recent experimental and human studies have generated the hypothesis that drugs that protect the vasculature and correct both arterial remodeling and endothelial dysfunction may result in better prognosis in hypertension. It has been demonstrated that treatment with ACE-Is[17] results in regression of small artery remodeling and endothelial dysfunction present in hypertensive patients. Mechanisms involved in these effects include, reduction in oxidative stress in the vascular wall, heart, and kidney; the decrease in cell migration and cell growth; diminished interstitial fibrosis; and an improvement in endothelial dysfunction.[17] All of these mechanisms and others are involved in some of the cardioprotective, vasculoprotective, and renoprotective actions of blockers of the RAS. These effects have been demonstrated in both experimental animals and humans and provide strong experimental support to the idea that these actions may improve prognosis in hypertension beyond BP lowering.

Recent experimental and human data suggest that ACE-Is reduce proliferations of vascular smooth muscle, enhance endogenous fibrinolysis, inhibit plaque rupture and vascular occlusion.[17] If this is true, can ACE-Is be used effectively in acute ischemic stroke as agents to improve stroke outcome? What is the current evidence in favor of their use in acute stroke?

## Angiotensin-I receptor antagonism

Angiotensin II (AT) acts by 2 types of receptors: the receptor (AT1), which mediates its actions on vasoconstriction, renin (inhibition), and aldosterone (stimulation) secretions, cellular proliferation and angiogenesis and the non-AT1 (often called AT2) receptors. Mainly expressed in the embryo, these latter may favor cellular differentiation and recruitment of collateral circulation. ACE-Is decrease the synthesis of AT, and therefore the stimulation of both receptor types, whereas AT1 receptor antagonists (AT1RAs) block only the stimulation of these latter and increase the stimulation of AT2 receptors because they increase the production of AT2. Experimentally, ACE-Is and AT1RAs decrease angiogenesis and cellular proliferation and favor cellular differentiation, which could explain the protection of ACE-Is against cancer. Despite their common suppressive effect on angiogenesis, AT1RAs may be better than ACE-Is to protect against ischemic events, especially the cerebral ones because they favor the rapid recruitment of collateral circulation.[18,19] This effect on collateral circulation would once again favor the use of AT1RA in acute stroke for favorable stroke outcomes.

Multiple factors are involved in thrombus formation. Platelet activation plays a crucial role in the genesis of acute coronary syndromes involving not only platelets but also endothelial cells, leukocytes, and erythrocytes. AT is a vasoconstrictor that could participate in the thrombotic process. Platelets also express AT1 receptors on their surface. Losartan is a nonpeptide blocker of AT 1 receptors. Since aspirin is now proven to improve stroke outcomes when administered as an antiplatelet in acute therapy for stroke, it may be argued that ACE inhibition/AT1RA blockade may have similar benefits through their actions on thrombus formation; fibrinolysis, plaque stabilization, and collateral circulation enhancement.

## ACCESS Study

The Acute Candesartan Cilexetil therapy in Stroke Survivors (ACCESS) study[6] was designed to assess the safety of modest BP reduction by candesartan cilexetil in the early treatment of stroke. The study was also designed to provide an estimate of the number of cases required to perform a larger phase III efficacy study. A total of 500 patients were recruited in a prospective, double-blind, placebo-controlled, randomized multicenter phase II study. The target reduction in BP was 10%–15% within 24 h using 4–16 mg of candesartan cilexetil. The primary end point was defined to include case fatality and disability measured as functional status with the use of the Barthel Index, 3 months after the end of a placebo-controlled 7-day phase. The 7-day course of candesartan after an acute ischemic stroke, significantly improved cardiovascular morbidity and mortality. The underlying mechanisms explaining this benefit are still unresolved. AT principally affects vascular tone and structure via two different pathways: vasoconstriction, which in turn releases inositol triphosphate and transmembrane Ca^2+^ influx, respectively. Vascular growth and the role of AT in vascular remodeling are known to be mediated via a pathway starting with the activation of the tyrosinase kinases, stimulation of the small G-protein Ras, activation of the serine/threonine kinase Ras-1, and subsequently the threonine/tyrosine kinase and the mitogen-activated protein kinases ERK1/2. The favorable effects of the early AT receptor blockers (ARBs) are mainly due to a lower incidence of myocardial ischemic events. Although the mechanisms by which ARBs affect cardiovascular morbidity and mortality are still unresolved, the ACCESS study demonstrated that early neurohumoral inhibition has similar beneficial effects in cerebral and myocardial ischemia.

A recent study aimed to compare stroke severity between stroke patients who were taking ACE-Is before their stroke onset and those who were not, to examine the effects of pretreatment with ACE-Is on ischemic stroke severity.[20] The study was to elucidate if ACE-Is have potential neuroprotective effects. Their results suggested that ACE-Is may reduce the clinical severity of stroke as measured by NIHSS score. In this study, there was no difference in admission BP between ACE-I and non-ACE-I users, suggesting that the beneficial effects of ACE-I use may not be directly related to their BP lowering effect. This is concordant with the results obtained by the HOPE trial.

A recent prospective observational study of 507 patients with first-ever ischemic stroke showed that treatment with ACE-Is at the time of stroke onset is associated with reduced plasma concentration of C-reactive protein and better long-term outcomes, suggesting that ACE-Is may have antiinflammatory properties and reduce the acute-phase inflammatory response after stroke onset.[21]

There are several other potential mechanisms by which ACE-Is may provide benefit to stroke patients. Experimental data suggest that the RAS modulates the atherosclerotic process, and that AT exerts proinflammatory actions in the vascular wall, which induce the production of reactive oxygen species and hydroxyl radicals, cytokines, and adhesion molecules.[22–25] ACE-Is could provide neuroprotection via blockade of AT-mediated endothelial dysfunction, lipid peroxidation, and subsequent oxidative stress and vascular smooth muscle intracellular calcium accumulation and hypertrophy.[26–27]

Furthermore, ACE-Is may help maintain hemostatic balance of fibrinolytic and procoagulant factors and increase cerebral blood flow.[28–29]

Recent studies using transcranial Doppler have shown that perindopril can improve the cerebral vasomotor reactivity in patients with lacunar infarcts beyond any effect on BP[30] and that treatment with quinopril can ameliorate cerebrovascular reactivity caused by methionine-induced hyperhomocysteinemia in healthy volunteers.[31]

Since a mild/moderate reduction in BP was also associated with reduction in stroke recurrence and since numerous non-BP lowering effects of ACE inhibition may translate into antithrombotic/fibrinolytic and plaque stabilizing properties, besides actively enhancing the collateral circulation through increased AT2 receptors, there is adequate scientific basis to test these drugs as agents modifying stroke outcomes. Future may envision trials with these drugs as therapeutic agents modifying the stroke outcomes.
